# Present and Future Applications of High Resolution Mass Spectrometry in the Clinic

**DOI:** 10.15190/d.2014.9

**Published:** 2014-06-30

**Authors:** Christopher A. Crutchfield, William Clarke

**Affiliations:** Department of Pathology, Johns Hopkins University School of Medicine, Baltimore, MD, USA

**Keywords:** High Resolution Mass Spectrometry, proteomics, clinical and forensic toxicology, microbiology, molecular diagnostics

## Abstract

High resolution mass spectrometers have directly enabled clinical applications of high clinical utility. These types of mass spectrometers are less known to the general public than their low resolution counterparts and are often ascribed to proteomics or biomarker discovery. This perception is rapidly changing as high resolution mass spectrometers see impact in the areas of clinical toxicology, forensic toxicology, microbiology, and molecular diagnostics as routine analyzers. Applications in these areas are made possible by the unique capacity of high resolution mass spectrometers, typically time-of flight or Orbitrap instruments, to characterize analytical species with sufficient mass resolution to better resolve molecular composition than lower resolution analyzers. This capacity confers a unique source of analytical specificity. In the future, this analytical specificity will likely be well applied to other clinical applications: mass spectrometry based tissue imaging, intraoperative determination of tumor boundaries, and evaluation of metabolic flux.

## INTRODUCTION

**Figure 1:**
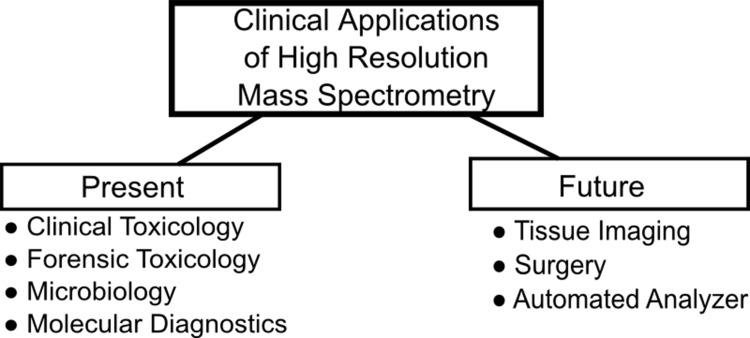
An outline of present and potential future applications of high resolution mass spectro-metry in the clinic

Over the past decade mass spectrometry has grown out of its roots as an analytical method relegated to niche research or industrial applications to a method routinely applied in clinical settings. The clinical setting requires accurate and robust measurement of diverse sets of analytes from a variety of matrices. The adoption of mass spectrometry in the clinic has certainly been slowed by the availability of large-scale immunoassay analyzers that could provide thousands of results on several analytes per day. Immunoassay based platforms have historically been more sensitive than mass spectrometers, though in recent years this divide is closing and in some cases mass spectrometry has surpassed immunoassays in this respect^1^. On the other hand, there is a greater likelihood of analytical interference in immunoassay-based analyzers than mass spectrometers. This has caused some professional societies, such as the Endocrine Society, to mandate that in the near future steroid hormone levels be generated from a mass spectrometry based analyzer for consideration for publication in the Society Journal^2^. This mandate comes at a time when the mass spectrometer has started to see considerable adoption in several clinical fields: small molecule analysis (e.g. toxicology), microbial identification, and molecular diagnostics. However, these fields may have not realized the clinical utility of mass spectrometry if not for the advent of *high resolution *mass spectrometry (HRMS)^3,4^. This review will briefly detail the analytical characteristics and performance of HRMS relative to conventional mass spectrometry. It will also discuss current clinical applications of HRMS as well as exciting new future applications in development. An outline of the present and future applications discussed herein can be found in **Figure 1**.

## HRMS Fundamentals 

Before discussing the applications of HRMS it is important to emphasize how and why the capacity to measure the mass-to-charge ratio (*m/z*) of ions in high resolution is important analytically. First, the utility of a mass spectrometer is based on its ability to distinguish molecules based on the mass and the charge of the ions they generate in an ion source. The principle that makes HRMS compared to conventional “unit” resolution mass spectrometry *really *useful is rooted in atomic physics with regards to the distinct nuclear binding energy of each isotope of every element, the *mass defect*^5^: the difference between the exact mass and nominal mass. With carbon-12 set at 12.00000 Da (where Da = Daltons, units designated one twelfth the mass of carbon-12), the exact mass of nitrogen-14 is 14.00307 Da. The mass defect for nitrogen-14 can then be calculated as 14.00307-14.00000= 0.00307. The exact masses, mass defects, and relative abundance for the most pertinent isotopes of common organic elements are in **Table 1**. It is important to note that in mass spectrometry when referring to elemental isotopes that they are typically *stable *isotopes—hence when a method is developed that utilizes an isotopically-labeled analogue of an analyte the heavy are C-13, not C-14; hydrogen atoms are typically replaced with deuterium, not tritium. However, as is listed in **Table 1** there is a natural abundance of heavy stable isotopes for a given element and that abundance will determine the isotopic distribution. Mass spectra can be difficult to interpret. Their interpretation is best facilitated when considering both the calculated mass defect of a peak as well as the presence of other peaks nearby. Interferences observed in method development may be part of the isotopic cluster of a compound with a different *monoisotopic mass*- the mass of a molecule that consists of the most abundant isotopic species of each element (e.g. C-12, H-1, N-14, etc.). The analytical consequence of each elemental isotope having a slightly different mass defect is that if the* m/z *of an analyte can be accurately measured, then the exact molecular formula can be determined computationally, provided the measurement was done with sufficiently high resolution. This is a distinct mechanism for analytical specificity from tandem mass spectrometry (MS/MS), whereby specificity is derived via an analyte-specific fragmentation event - in the clinic these instruments are typically triple quadrupole mass spectrometers.

**Table 1:** Exact Masses of Common Organic Isotopes and their mass defect

Isotope	Exact Mass (Da)	Mass Defect (Da)	Isotopic Composition
^1^H	1.007825	0.007825	0.999885
^2^H	2.014102	0.014102	0.000115
^12^C	12.000000	0.000000	0.9893
^13^C	13.003355	0.003355	0.0107
^14^N	14.003074	0.003074	0.99636
^15^N	15.000109	0.000109	0.00364
^16^O	15.994915	-0.005085	0.99757
^18^O	17.999161	-0.000839	0.00205
^31^P	30.973762	-0.026238	1.0000
^32^S	31.972071	-0.027929	0.92223

**Figure 2:**
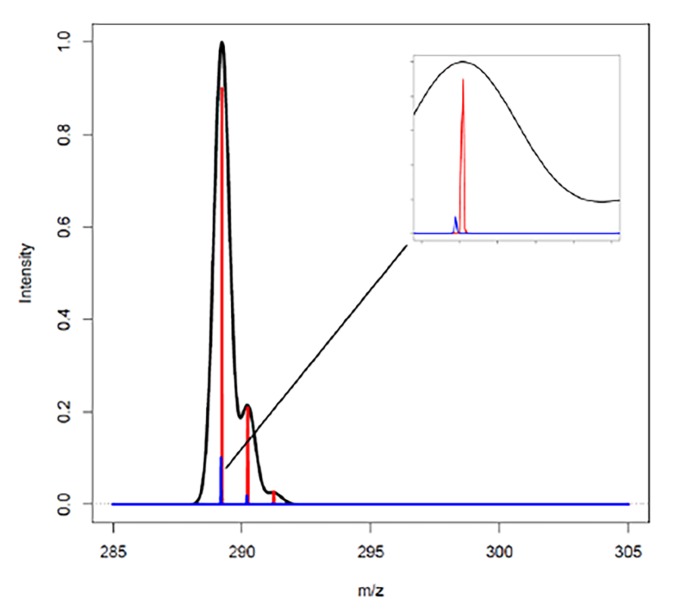
A simulated spectrum showing the mass resolution of testosterone (red) and estriol (blue) with high resolution that could not be resolved using an instrument with low resolution (black). The low resolution instrument combines signals from both components to make it appear as a single peak because both compounds have the same nominal protonated mass of 289 Da. The simulated high resolution traces have a resolution of 15,000 at 300 Da. Here the two analytes are clearly defined as different chemical components of the mixture.

There are two related terms used to describe the performance of a high resolution mass spectrometer: mass resolution and mass-resolving power. They are often used interchangeably though they have very specific IUPAC definitions^6^. The peak width definition for resolution is:

*r = m/ (∆m)* (1.1)

where *m *is the mass of a single peak in a mass spectrum and *Δ*m is the width of the peak at 50% of maximal peak height, or full width at half maximum (FWHM). The resolution is dependent on the mass at which it is measured therefore the mass should always be stated in the context of resolution. The exact dependence of resolution to mass is different for different types of mass analyzers. TOF analyzers have relatively constant resolution for *m/z* while Orbitrap analyzers have relatively lower resolution at high *m/z*. A simulated spectrum is presented in **Figure 2** that demonstrates the mass resolution of two steroid hormones testosterone and estriol using high resolution instrumentation that would have the same nominal mass. This illustrates the mechanism HRMS uses to achieve high analytical specificity. This specific example leveraged the differences in the mass defects of carbon, hydrogen, and oxygen to distinguish the two molecules.

Generally, high resolution mass spectrometry implies *accurate *mass measurements in addition to resolution. However, these parameters are mutually exclusive: different mass spectrometers address these issues, that of resolution and accuracy, differently. The definition of mass accuracy is usually given in parts per million (ppm):

*ppm = 1.0 x 10^6^ (measured mass-theoretical mass)/theoretical mass * (1.2)

High mass accuracy is achieved by operating an instrument with high resolution as well as having the instrument sufficiently calibrated. An ideal instrument would have zero systematic bias due to insufficient calibration or drift. In this circumstance the accuracy of the instrument would simply reflect the precision of the mass analyzer. To approach this ideal scenario high resolution mass spectrometers require periodic calibration—ideally via internal calibration and external calibration strategies. External calibration is the determination of parameters used to calculate mass external to the mass peak in a spectrum. Internal calibration uses mass peaks *within* a spectrum to mass correct. Internal calibration typically improves mass accuracy ~2-fold relative to external calibration^3^.

Clinical application of high resolution mass spectrometry has been primarily limited to Time-of-Flight (TOF) mass spectrometers and Orbitrap mass spectrometers. The instruments operate on different technical principles to attain high mass resolution measurements. TOF instruments attain high mass resolution by precisely measuring the time it takes for ions to traverse a flight tube and hit a detector. TOF instruments are extremely fast at scanning a large range of masses. They also maintain higher resolution at higher *m/z *values (>~2000 Da) relative to Orbitrap mass spectrometers. Orbitrap mass spectrometers generate high resolution mass measurements by detecting the axial frequency of an ion as it oscillates inside the trap. Orbitrap technology has only been recently developed, and was commercialized in 2005. It has gained substantial popularity for its ability to provide mass resolution much greater than TOF instruments, particularly at lower *m/z* (~<1,000 Da). It should be noted that the capacity to generate very high resolution mass measurements comes at the expense of scan speed. However, with the widespread adoption of ultra-high performance liquid chromatography analyte peak widths have been reduced to seconds—this forces a compromise between determining a high resolution mass measurement or comprehensive chromatographic peak detection.

Notably, both conventional triple quadrupole MS instruments and hybrid HRMS instruments provide front-end ion optics to operate in MS/MS (product ion mode) mode. High resolution mass spectrometry alone does not differentiate isobaric (equal mass) analytes. Analytical strategies that assist in isobaric analyte separation are chromatography or MS/MS fragmentation. These strategies are often employed even in the absence of isobaric interference in order to improve analytical specificity. There are other acquisition modes in which a mass spectrometer can be operated in addition to product ion mode that include neutral loss scan and precursor ion scan among others^7^. These acquisition modes are not routinely used in the clinic, but are worth mentioning because they have been useful some class specific analyses, such as phospholipids^8^. Full scan commonly denotes using HRMS to analyze the signal intensity from all ions generated in the ion source. This acquisition mode is ubiquitous from HRMS because it has much greater analytical selectivity compared to full scan acquisition using a lower resolution instrument.

All clinical mass spectrometry, whether performed with high resolution or not, uses instrumentation that generates ions from the analyte(s) of interest. Depending on the application, contemporary ionization processes typically arise from either the liquid phase or the solid phase. Ionization of analytes from the liquid phase is typically accomplished via electrospray ionization (ESI), atmospheric pressure chemical ionization (APCI), or atmospheric pressure photoionization (APPI) in concert with liquid chromatography^9,10^. These ionizations sources have differential ionization efficiency for analytes depending on analyte polarity: ESI has the greatest efficiency at ionizing polar molecules and APPI being most adept at ionizing nonpolar molecules, though these effects are high analyte specific. Alternatively, ions can be generated from the solid phase via matrix-assisted laser desorption ionization (MALDI)^11^. Other techniques for generating ions exist, however ESI, APCI, APPI, and MALDI methods are the most prevalent in a clinical setting. While the specific physical mechanisms by which these different ionization sources differ, they all operate on the principle of applying energy to the liquid or solid medium carrying the analyte which results ion generation, typically by the addition or subtraction of a proton. However, the ionization process is complicated and depending on the analyte, ionization source, and source conditions analytes can pick up multiple charge states, can fragment within the source, and can have other molecular features associated with their ion (e.g. sodium addition to form a sodium “adduct”).

## Clinical Toxicology

The toxicology lab is one area that has adopted LC-HRMS as a routine analytical methodology. Prior analytical methods that were historically widely used, such as GC-MS are labor intensive, and rely on analytes amenable to GC analysis (principally, analytes that are volatile or can be chemically modified to become volatile). LC-MS/MS methodology requires careful methodological design to generate analyte specific fragmentation products to monitor. LC-HRMS has helped alleviate the pitfalls of previous methods. As an LC based methodology, analytes typically do not require chemical derivatization for their analysis. Nonetheless, with high resolution accurate mass, the instrument can provide exquisite analytical specificity for the presence or absence of toxicological substances present in a biofluid. Most analytical workflows depend on an instrument-specific accurate mass toxicological database of known substances. After analysis of a specimen, the database will match mass peaks with its values and provide a list of putative matched compounds. More specific strategies for toxicological substance detection use more a traditional “targeted” approach whereby retention time windows are identified in which known analytes elute. Using stable-labeled internal standards as an internal QC metric, the presence of analytes are qualitatively determined using an intensity cutoff. HRMS applications to toxicology have not been limited to either Orbitrap or TOF based technology. As mentioned earlier, the choice between technologies is highly dependent on the specific application^12^.

One appealing feature of HRMS is the ease of assay development compared to conventional immunoassay approaches. Immunoassay method development takes considerable time to due to the process of developing an antibody with binding specificity for the analyte of interest. HRMS-based methods only require the drug (or its metabolite) for method development. HRMS enables rapid implementation of drug testing in clinical settings that involve patients consuming novel or uncommon toxic substances. The past decade has seen large growth in the abuse of designer drugs, particularly synthetic cannabinoids^13^ and stimulant cathinones, “bath salts”^14^. In 2012, Wu *et al*^15^ remarked on the lack of commercial immunoassays for these drugs. Specific immunoassays are remarkably difficult to design for these drugs because there are a large number of analogues. Kronstrand *et al *recently remarked on the superiority of a LC-Q-TOF analytical strategy over immunoassay for comprehensive analysis of synthetic cannabinoids in urine^16^. They remark that although an immunoassay test had reactivity with MAM-2201 and JWH-122 metabolites, it did not react with UR-144 metabolites. UR-144 metabolites were found in specimens analyzed and demonstrate the risk of false negatives when relying on immunoassay methods. In 2013, Concheiro *et al* developed the most comprehensive to-date quantitative screen of cathinones, measuring 28 synthetic cathinones in urine^17^. Notably, the authors used an Orbitrap with a quadrupole frontend that can operate in MS/MS mode.

## Forensic Toxicology

A significant appeal of HRMS based detection in toxicological settings is the ability to perform retrospective analysis when full-scan data is obtained. Here, the data acquired during LC-MS acquisition would be archived after reporting the presence or absence of known substances. In 2013, Rosano *et al *describe a post-mortem drug screening method using UPLC-MS^E^-TOF that evaluated the presence of over *950 *toxicologically relevant drugs and metabolites^18^. In their study they claim a 99% detection rate using UPLC-MS^E^-TOF: a feature on Waters Q-TOFs (Waters, Milford, MA). A low resolution mass spectrometer only provided 80% detection rate. However, this particular study did not have positive controls for ~10% of the compounds screened. The remainder of the drugs were only determined via a database search of the MS^E^ data acquisition. MS^E^ is an example of data *independent *acquisition (DIA). DIA strategies acquire product ion information of ions independent of intensity. Different manufacturers use different DIA strategies. MS^E^ ramps collision energy and fragments *all *ions generated by the source, generating a convoluted product ion spectrum that results from all initial ions. Software algorithms process these data and attempt to deconvolute the spectra into individual features.

Alternative DIA strategies include SWATH which is featured on AB Sciex Triple-TOFs (AB Sciex, Foster City, CA), which generates convoluted spectra at variable (typically ~25 Da) isolation bins across a variable (typically ~600 Da) window. These data are empirically less convoluted than the MS^E^ data because the ions inserted into the collision cell consist of a few ion species. These strategies have been developed in response to data dependent acquisition strategies (DDA), which operate on the basis of fragmenting ions on the priority of ion intensity. The issue with DDA is that low intensity ions, though they may be clinically significant, may not be fragmented and generated into a feature. Database-driven toxicology is problematic. Even with extraordinarily good spectral match scores, the possibility of a false positive is significant. The niche where database-driven toxicology has a future is in hypothesis generation for a drug confirmation. If a compound is putatively identified by fragmentation, the analyst can perform a spike-recovery experiment using the reference compound. In the future, in combination with a standard mixture of isotopically labeled internal standards, a standard UPLC method may produce a sufficiently inter-lab reproducible retention times to approximate a retention-index-like parameter to combine both MS/MS and retention time to better predict analyte identity using a single database.

Marzinke *et al*^19^present an application of HRMS screening of antiretroviral (ARV) drugs for evaluating eligibility in a HIV clinical trial. The study examines a difficult consequence of HIV clinical trials: trials commonly attempt to recruit newly diagnosed, drug naïve patients; however, patients may be untruthful with regards to their knowledge of their HIV status as an attempt to obtain care by enrolling in the trial. The assay was a qualitative screen analyzing for the presence of 15 ARV drugs in patient plasma using liquid chromatography coupled to an Orbitrap Mass Spectrometer. Their study found that of the 155 newly diagnosed HIV positive patients, 45.8% had at least 1 ARV drug detected during their enrollment period—almost half on the enrollees were not drug naïve! This study provides a framework for establishing analytical prerequisites for clinical trials of chronic conditions where rejection criteria include previous exposure to a drug. This analytical challenge will only become more difficult as the general population begins to understand the power clinical labs now in screening for non-illicit drugs in a study setting.

## Microbiology

HRMS alone, principally in the format of matrix assisted laser desorption ionization time-of-flight mass spectrometry (MALDI-TOF) has shifted the microbial screening paradigm from antiquated phenotypic algorithms to a cost effective, rapid, and accurate mode of microbial species identification^20^. These classical methods included staining, culturing, biochemical testing, and susceptibility testing. Some analyses could last weeks. The MALDI-TOF workflow has unified testing strategies across several classes of microbes. Tan *et al* evaluated the cost and time savings from adopting MALDI-TOF and found on average identification was produced 1.45 days earlier and at roughly half the cost, with the majority of those savings in reagent costs^21^. Huang *et al* found institutional and society cost savings are even further amplified when considering implementation of an antimicrobial stewardship team using MALDI-TOF data can drop the length of intensive care unit stays from 14.9 to 8.3 days^22^.

Depending on the cell wall strength of the microbial class, a colony will be placed on a MALDI target directly, after extraction with acid, or after bead lysis. The preparation is finished with an overlay of chemical matrix, typically 4-α-cyano-4-hydroxycinnamic acid (CCA). The target is irradiated by a laser and mediates the generation of protonated gas ions of polypeptides and small proteins—the majority of which are ribosomal or DNA binding in origin. The spectra generated are computationally scored against reference spectra in a database supplied with the instrument and a report is generated providing the confidence of microbial species identification. Because these scores have confidence intervals generally defined as species, genus, and indeterminate—the microbe can be reflexed to a more sensitive method in circumstances when the MALDI-TOF fails to provide an accurate identification. Interestingly, an alternative to the MALDI-TOF analysis of proteins from microbiological specimens has been to use ESI-TOF analysis of nucleic acids. This technology will be discussed in greater detail in the molecular diagnostics section.

There are two major platforms for MALDI-TOF microbial identification analyzers: the Bruker Biotyper (Bruker Daltonik GmbH, Bremen, Germany) and the bioMérieux Vitek MS (bioMérieux, Marcy l’Etoile, France). In a direct comparison both devices performed well in accurate identification of microbes, with genus agreement >~90% and species agreement >~70%^23^. Both instruments have their own databases. These databases can be periodically updated to include additional organisms and improve overall accuracy. The most compelling aspect of these instruments for the clinical laboratory is how relatively little training is required to operate the instrument. Because the interpretation of the microbes is automated via the computer the most major concern is appropriate sampling and preparation of the specimens.

## Molecular Diagnostics

Mass spectrometers as detectors for nucleic acids have been in the shadow of massively parallel next generation sequencers (NGS), which conventionally have light-based or electrochemical based detectors. However, nucleic acid mass spectrometry does fulfill its own broad, clinically relevant, niches: microbial identification^24^, microbial susceptibility testing, viral identification, SNP genotyping, methylation analysis, gene expression analysis, copy number variation, and comparative sequencing^25,26^. All of these applications rely on polymerase chain reaction (PCR) amplification of targets sequences. As adoption of these technologies in the clinic are still seeing their utility grow and the direct costs of NGS technology and mass spectrometry technology continue to become amenable, we will not directly compare the two. However, it is worthwhile to emphasize that NGS sequencers can only analyze nucleic acid sequence. Mass spectrometry as a technology enables the analysis of broad classes of clinically relevant analytes including but not limited to nucleic acids. Due to this analytical flexibility an automated analyzer may eventually be developed that replaces the immunoassay. This analyzer would use mass spectrometery as the detector and would provide routine testing capabilities in the clinical applications of toxicology, endocrinology, microbiology, and molecular diagnostics. Due to the high potential of this technology of HRMS assuming a central role in the laboratory it is important to discuss all its clinical applications, including molecular diagnostics.

Nucleic acid mass spectrometry has been predominantly commercialized via the Sequenom MassARRAY^27^and the Ibis T5000^28^platforms. These two platforms differ substantially in how they analyze the nucleic acid molecules. The Sequenom platform uses MALDI-TOF and the Ibis platform uses ESI-TOF. In addition, the sample preparation for the two methods differs slightly in that the Sequenom platform operates on the principal of primer extension and the Ibis platform operates on the principal of “Triangulation Identification for the Genetic Evaluation of Risk (TIGER)”^28^. The analytical measurements the instruments make are the same-- that is, measuring the *m/z* of an amplified product. However, the Sequenom platform is measuring the mass addition of a primer extension product and the Ibis platform is measuring the *m/z *of several amplicon products. The Sequenom platform result gives true “sequencing” data—that is, the difference in *m/z* corresponds to the base added during primer extension. Conversely, the Ibis platform never provides sequence information. Rather, it relies on the accuracy of the *m/z *measurement to determine amplicon base composition. The amplicon base composition is used in a calculation to determine the origin of the DNA, typically a microbe or a virus.

The majority of the infectious disease field has focused on the capacity of mass spectrometry to impact microbial identification. However, viral infections are extraordinary important from both a clinical and epidemiological standpoint. The standard MALDI-TOF based platforms for microbial identification require colony isolation before identification. Some infectious disease agents are more appropriately identified using direct sampling—e.g. virus or difficult to culture microbes. This workflow has been commercialized as PLEX-ID using the Ibis platform. PLEX-ID takes advantage of PCR to amplify relatively low concentrations of nucleic acid to detectable concentrations by mass spectrometry. Another benefit to this approach is the selectivity afforded by the PCR reaction, which limits the potential amplicon products that will be analyzed by the mass spectrometer. With carefully designed sequence primers, subtyping has become possible. Tang *et al* reported on the accuracy of the influenza A and B assay, PLEX-ID flu^29^. They found that the overall accuracy when evaluating the presence of either influenza A or B and if found to be influenza A subtyped as H1N1-p, H1N1-s, or H3N2 compared to a RT-PCR based assay Prodesse ProFLU+ for Influenza A or B typing, and Prodesse ProFAST+ for A subtyping to be 97.1-100%.

The Sequenome platform provides a flexible platform for high throughput genotype screening. After primer extension and target preparation, the nucleotide analysis is very rapid due the MALDI ionization mechanism. Moreover, as the specimen preparation utilizes PCR the assay is not limited by analytical sensitivity, which sometimes is a challenge for MALDI-based methods. The device has seen application in screening for SNPs, such as in a study done by Sinotte *et al* evaluating mutations in Vitamin D binding protein and their consequence in plasma concentrations of 25-hydroxy vitamin D in premenopausal women^30^. Godfrey *et al* use the Sequenome platform for assessing methylation status of five candidate genes and associated them with risk for adiposity^31^ is work has contributed to the epigenetic component of risk for metabolic disease and obesity. Holzinger *et al* have demonstrated the utility of the Sequenome platform for evaluating CYP2B6 polymorphisms associated with efavirenz pharmockinetics^32^.

## Future HRMS Clinical Applications & Developments

The role of mass spectrometry in the clinic is growing. While the past decade has seen many new applications grow into fruition, several other technological developments are developing that can change the way patients have a diagnosis determined or have an operation performed. Both of these applications are a consequence of direct tissue sampling for mass spectrometric analysis. The first application has been posited as the beginning of a new era for histology: mass spectrometry imaging (MSI). MSI attempts to molecularly characterize a tissue in micron resolution by precisely ionizing an area of a tissue and recording the relative intensities of the ions generated^33-36^. MSI has been conventionally been performed using MALDI, however other ionization mechanisms have been applied for the same purpose^37^. MSI is typically performed using direct ionization of analytes in tissue. Like microbial identification, it relies fully on the quality of the mass spectrum to make chemical assignments. As a result, utilization of HRMS for the MS acquisition in a MSI application can improve analytical specificity. The primary challenges of MSI are the cost (traditional staining strategies are routine in the clinical setting), the time for analysis (an image may take several hours for its acquisition), relative lack of spatial resolution (laser spot sizes can be as large as several 100 μm in diameter), and ability to generate reliably quantitative information on analyte concentration. Nonetheless, clinical applications have been developed that could supplant traditional approaches when the clinical utility of multi-marker analysis has been demonstrated. For example, Rauser *et al*^38^ demonstrate the analysis of HER2 receptor status in breast cancer tissue using MALDI MSI. In the future MSI data may be used to direct treatment and resection of a tumor—for example, Li and Hummon have demonstrated the application of MSI to analyze the relative distribution of proteins in a carcinoma spheroid in an attempt to better characterize the effect of differential hypoxia on tumor expression^39^.

Another exciting new application that could be adopted by the HRMS field is Rapid Evaporative Ionization Mass Spectrometry (REIMS). REIMS takes advantage of an existing byproduct of electrosurgical devices, “smoke,” to identify if the tissue origin of the smoke is cancerous^40^. This is made possible by the molecular ion formation in the smoke discharge when tissue is excised using an electrocautery device. Mass spectra are acquired throughout the surgery and input into a multivariate classification model to determine similarity with spectra from a known database. Balog *et al*^40^ used a moderate resolution mass spectrometer for their initial *in vivo *component of the report using an LCQ Deca XP Plus. However, their *ex vivo *work utilized an Orbitrap Discovery mass spectrometer with >10,000 FWHM. Their classification rates were very good, with misclassification rates depending on tissue type between 0-7.7%. The only analytical separation during the analysis is via the time and space dimensions of the usage of the electrocautery device and the mass differences observed in the mass spectra. Without a physical separation the analytical determination relies solely on the quality of the mass spectra. The classification rate of this method might further improve with a higher resolution mass spectrometer driving the analysis.

The technology driving MS-based clinical analysis is still being rapidly developed. One development that has future promise for radically changing LC-MS throughput is ion mobility spectrometry (IMS). IMS technology is based on the principle of moving ions through a drift tube with a carrier buffer gas. This gas resolves ions based on their size and shape - as a result larger molecules, those with greater collisional cross section, have longer drift times. This separation occurs on the millisecond time scale. Unfortunately, the IMS resolution provided is typically only sufficient to resolve ions of different chemical class or charge state - a good application of this has been demonstrated by Jackson *et al* in their ion mobility MSI analysis of lipids in rat brain tissue^41^ as a method of isolating lipids ion species from peptides and matrix. With more development, particularly in the areas or drift cell design and carrier gases, we may see applications that require ultra-fast analysis supplant traditional LC-ESI-MS approaches with ESI-IMS-MS. Combined with a high resolution mass spectrometer a fully realized implementation of this technology would absolutely resolve the molecular formula of ions as well as fully resolve them from isobaric species in seconds. At the moment throughput is routinely increased by multiplexing parallel liquid chromatography modules to facilitate mass spectrometric detection at only the point of analyte elution^42^. LC systems require maintenance and have associated consumable costs in the form of chromatography columns and solvent. If IMS could replace LC it would reduce costs associated with the maintenance and consumables.

Although it is too early to establish clinical utility- a biological application uniquely suited for high resolution mass spectrometry is metabolic flux analysis^43,44^. This analysis investigates differential utilization of biochemical pathways, typically in a cell line. When applied to cancer, for example when using an isotopically labeled glucose or glutamine, aberrant increased production of lactate (Warburg effect) from glucose or production of fatty acids from glutamine can be seen. HRMS facilely enables the differentiation of the isotopomer species of these analytes. The research is still so early in development it is difficult to ascertain the clinical implications of subtyping cancers on differential metabolic flux analysis^45,46^. However, there may be therapeutic interventions that apply strictly to cancers that have reorganized their glutamine metabolism versus those that have not based on drugging different targets^47^. These interventions could be implemented as a personalized approach to cancer therapeutics. The benefit to this type of analysis relative to a molecular test is that the result could have an established reference range based on flux—it could be independent of an individual cancer genotype. This is in contest with next generation sequencing assays that rely on databases to establish if a molecular variant is associated with malignancy. This analysis would also finally close the diagnostic loop. PET scanning often relies on consumption of 18fluorodeoxyglucose (18F-FDG) to accumulate at the site(s) of cancer. However, the metabolic fate of the increased glucose influx is never determined. HRMS may eventually determine this metabolic fate in order to direct clinical decision-making.

Mass spectrometry is a powerful analytical tool because of its capacity to analyze molecules independently of their size and class. Recently, Kullolli *et al* reported on the analysis of MicroRNAs (miRNAs) using HRMS^48^. miRNAs are short non-coding RNAs that regulate gene expression. They have been suggested as possible biomarkers for disease, as they have extracellular circulation in the blood. Creemers *et al* discuss their possibility as putative cardiovascular biomarkers^49^. This is an example of the flexibility of HRMS adapting to the molecules of high clinical interest for diagnostic purposes.

## CONCLUSIONS

HRMS has started to find its way into the clinical laboratory. Not only has it been established as a viable alternative to traditional triple quadrupole mass spectrometer based quantification, but has enabled application developments that would have been impossible to develop without it, such as microbial identification or molecular assays. The versatility of HRMS, especially in the context of more conventional availability of hybrid-HRMS instruments, challenges the paradigm that all clinical mass spectrometers must be low resolution and base their analytical specificity on tandem mass spectrometry. The widespread adoption of HRMS and mass spectrometry in general will certainly fuel more technological developments. Research well focused on providing technical solutions to addressing the unmet needs of mass spectrometry based analyzers, specifically with trying to address issues of analytical sensitivity, such as the development of the ion funnel which increases sensitivity by approximately an order of magnitude^50^. Greater analytical sensitivity may generate opportunities to investigate novel analytes of clinical significance. With additional developments in quantification strategies the high resolution mass spectrometer is positioning itself into a space where it could be a new cornerstone in all areas clinical decision making: a device that at the point of care simultaneously determine all toxicological and hormonal disorders presenting in a patient, in the microbiology lab will determine the identification and drug susceptibility of infections, and in the surgical suite help elucidate tumor margins during resection. These applications and more will make for a very exciting next decade of clinical mass spectrometry.

- ***High Resolution Mass Spectrometry (HRMS)’s*** **analytical selectivity, due to accurate estimation of molecular mass, enables unique clinical applications, such as: the rapid identification/analysis of microorganisms, mass spectrometry imaging, and retrospective drug analysis. **
- **In the future *HRMS* may be able to serve the role as a universal detector for the majority of analytes measured in the clinic.**
